# Intravenous Thrombolysis Combined with Arterial Thrombolysis (Bridging Therapy) Effectively Improves Vascular Recanalization Rate in Patients with Cerebral Infarction

**DOI:** 10.1155/2022/8295212

**Published:** 2022-07-26

**Authors:** Junting Huo, Wei Li, Yu Liu

**Affiliations:** Department of Neurology, Beijing Chuiyangliu Hospital, Beijing 100022, China

## Abstract

**Objective:**

To explore the efficacy of intravenous thrombolysis combined with arterial thrombolysis (bridging therapy) in patients with acute cerebral infarction and its effect on serum inflammatory factors.

**Methods:**

The case data of 138 patients with acute cerebral infarction admitted to our hospital from February 2019 to February 2021 were retrospectively analyzed. According to the treatment plan they received, patients were assigned to two groups, namely, an observation group (*n* = 71) treated with bridging therapy and a control group (*n* = 67) treated with intravenous thrombolysis alone. The following indexes were recorded and compared between the two groups: treatment efficacy, National Institutes of Health Stroke Scale (NIHSS) score, activities of daily living, incidence rates of vascular recanalization, intracranial hemorrhage and reembolization after treatment, levels of inflammatory factors before and after treatment, levels of prothrombin time (PT), activated partial thromboplastin time (APTT) and fibrinogen (FIB) before and 1 week after treatment, and modified Rankin Scale scores 1, 3, and 6 months after treatment.

**Results:**

Compared with the control group, the therapeutic efficacy, neurological function, activities of daily living, and vascular recanalization were markedly better in the observation group (*P* > 0.05). In addition, the incidence of intracranial hemorrhage and reembolization was statistically lower in the observation group (*P* < 0.05). No marked difference was found between the two groups in terms of pretreatment serum inflammatory factors and coagulation function (*P* > 0.05), while the above indicators improved statistically after treatment in both groups, with comparatively more obvious improvement in the observation group. It was also observed that, compared with the control group, the modified Rankin Scale score in the observation group was significantly better at 3 and 6 months after treatment (*P* < 0.05).

**Conclusion:**

Bridging therapy can improve the vascular recanalization rate among patients suffering from acute cerebral infarction, reduce the incidence of intracranial hemorrhage and reembolization, and improve the prognosis and neurological function of patients, which is worthy of clinical application.

## 1. Introduction

Cerebral infarction refers to a clinical syndrome in which cerebral blood vessels are blocked due to multiple reasons, inducing hypoxic necrosis and ischemia of local brain tissue and, consequently, corresponding neurological deficits [1]. Characterized by a sudden onset with high morbidity, mortality, and disability rates, acute cerebral infarction (ACI) is the main disease causing death and disability of human beings, with a mortality rate of about 5-15% in its acute phase. Most patients will have sequelae, which not only endanger the safety of the patient but also cause a burden to the patient's family and to society [2, 3]. Therefore, reducing the fatality and disability rate of ACI is an urgent problem that needs to be solved. And to this end, opening the occluded blood vessel as quickly as possible is also essential, as well as restoring cerebral blood flow for ischemic penumbra tissue and reducing final infarct size, all of which will ultimately contribute to improved clinical outcomes of ACI patients [4].

Clinically, ACI patients are mainly treated by thrombolytic therapy, including intravenous thrombolysis, arterial thrombolysis, and arteriovenous combined thrombolysis. Among them, intravenous thrombolytics such as urokinase and recombinant tissue plasminogen activator (rt-PA) are widely in clinic due to their advantages of simple operation, rapid recanalization of cerebral blood vessels, fast restoration of blood flow, and preservation of neurological function of brain tissue within the onset time window [5, 6]. However, thrombolytic therapy has a low vascular recanalization rate and insignificant therapeutic effect on patients with refractory emboli or severe cerebral artery stenosis [7], while arterial thrombolysis, with the rapid development of interventional techniques, has been confirmed to render significant benefits to patients with large vessel occlusion [8]. Bridging therapy has also been increasingly used clinically in recent years, as it has a high vascular dredging rate and can quickly restore cerebral blood perfusion [9]. However, there are few studies on the efficacy of intravenous thrombolysis combined with arterial thrombolysis (bridging therapy) in patients with ACI.

In this study, 138 ACI patients admitted from February 2019 to February 2021 were enrolled to analyze the efficacy of bridging therapy (intravenous thrombolysis combined with arterial thrombolysis), in the hope of providing more clinical treatment references for the treatment of ACI.

## 2. Materials and Methods

### 2.1. Clinical Information

The case data of 138 ACI patients admitted to our hospital from February 2019 to February 2021 were retrospectively analyzed, including 75 males and 63 females with an average age of 65.34 ± 3.13 years. The study subjects were assigned to 2 groups according to different treatment plans, namely, an observation group (*n* = 71) treated with bridging therapy and a control group (*n* = 67) treated by intravenous thrombolysis alone. Inclusion criteria are as follows: (1) patients who met the diagnostic criteria for ACI [10], (2) patients with initial onset, (3) patients with a duration of less than or equal to 4.5 hours from symptom onset to intravenous thrombolysis, (4) patients with blood pressure < 180/100 mmHg, and (5) patients with no bleeding tendency and no recent history of major surgery. Exclusion criteria are as follows: (1) patients with intracranial hemorrhage and no early imaging changes of massive cerebral infarction; (2) patients with severe cardiac, hepatic, and renal insufficiency; (3) patients with previous intracranial hemorrhage and history of myocardial infarction and head trauma within the past 3 months; (4) patients with active bleeding and severe trauma; (5) patients with platelet count < 100 × 10^9^/L; and (6) patients with incomplete clinical data who refused to cooperate with the present study. The study has gained approval from the Ethics Committee of our hospital and complies with the Declaration of Helsinki.

### 2.2. Treatment Methods

The control group was given 0.9 mg/kg alteplase for injection (rt-PA, Boehringer Ingelheim Pharma GmbH & Co. KG, Germany) for intravenous thrombolysis, with the maximum dose of 90 mg. 10% of the total dosage was injected intravenously, and the rest was intravenously dripped within 60 min. Regular assessment of neurological function was then provided (once within 30 minutes in the first hour and once every hour thereafter until 24 hours).

In the case of hypertension, severe headache, nausea, or vomiting, thrombolytic drugs would be stopped immediately for brain CT examination. Regular monitoring of blood pressure, which was conducted once every 15 minutes for the first 2 hours, once every half an hour for the coming 6 hours, and once every hour thereafter until 24 hours, was also performed. And when systolic blood pressure ≥ 180 mmHg (1 mmHg = 0.133 kPa) or diastolic blood pressure ≥ 100 mmHg was observed, the number of manometry would be increased and antihypertensive drugs would be provided.

Patients in the observation group were treated with intravenous/arterial thrombolysis (bridging therapy). First, 10% of 0.9 mg/kg of alteplase (rt-PA) was injected intravenously. Then, the remaining dosage was pumped intravenously within 1 hour while sending the patient to the digital subtraction angiography (DSA) room. Under local anesthesia with lidocaine, an arterial sheath was placed through the right femoral artery to guide the tip of the instrument to the lesion site. The catheter was inserted with a guidewire, which was withdrawn before the catheter head was extended into the stent. The stent was then placed, and the thrombus was removed. After angiography, the stent was removed, and 30 mL of blood was drawn to avoid the regurgitation of prolapsed thrombus into the artery. After the success of thrombectomy shown by angiography, the arterial sheath was removed, the puncture point was bandaged, and the incision was closed. 24 hours after thrombolysis, patients with no obvious complications or intracerebral hemorrhage confirmed by head CT examination were given aspirin enteric-coated tablets (Bayer Health Care Co., Ltd.), 100 mg/d. For those with imaging findings of hemorrhage, antiplatelet and other antithrombotic therapy such as aspirin were prohibited, and instead, dehydration and intracranial pressure reduction and neuroprotective agents were given as appropriate according to the specific condition of each patient.

### 2.3. Observation Indexes

(1) After 24 hours of treatment, the clinical efficacy of two groups was recorded and evaluated. Specifically, it was divided into markedly effective (the patient's NIHSS score was reduced by >6 points, with markedly alleviated neurological dysfunction and effectively recanalized blood vessels), effective (the patient had a NIHSS score reduced by 3-6 points, basically alleviated neurological dysfunction and effectively recanalized blood vessels, and could work and live independently but with relative delay), and ineffective (the patient's NIHSS score was reduced by <3 points with obvious neurological dysfunction and blood vessel stenosis). Total effective rate = (markedly effective + effective) cases/total number of cases × 100%. (2) The National Institutes of Health Stroke Scale (NIHSS) [11] was applied to evaluate the neurological function of patients in the two groups before and 24 hours after treatment from the dimensions of visual field, facial paralysis, limb movement, etc., with a total of 42 points, and a higher score means poorer neurological function. (3) The Barthel index [12] was utilized to evaluate patients' activities of daily living before and after treatment from 10 domains of bathing, dressing, grooming, urination, defecation, toileting, bedding or wheelchair transferring, walking, and going up and down stairs, with a total score of 100. Higher scores suggest better activities of daily living. (4) Head CT examination was performed on patients in both groups after treatment, and the incidence rates of recanalization, intracranial hemorrhage, and reembolization were compared. (5) The levels of inflammatory factors of two groups, including interleukin-6 (IL-6), tumor necrosis factor-*α* (TNF-*α*), and high-sensitivity C-reactive protein (hs-CRP), were determined by enzyme-linked immunosorbent assay before and after treatment and compared between the two groups. (6) An automatic coagulation instrument was used to compare the levels of prothrombin time (PT), activated partial thromboplastin time (APTT), and fibrinogen (FIB) between the two groups before treatment and 1 week after treatment. (7) The modified Rankin Scale [13] was used to assess the prognosis of patients at 1, 3, 6, and 12 months after treatment with a total of 6 grades. The higher the grade, the worse the prognosis

### 2.4. Statistical Methods

SPSS 19.0 statistical software was applied for statistical analysis of the data, and GraphPad 7 was used for image rendering. Count data were recorded in the form of number of cases and percentage (%). Statistical analysis was conducted using the chi-square test. For measurement data, the intergroup comparison and intragroup comparison were performed by independent *t*-test and paired sample *t*-test, respectively, with *P* < 0.05 as the significance level.

## 3. Results

### 3.1. Comparison of General Information

The two groups of patients were comparable as there were no marked differences in gender, age, BMI, etc. (*P* > 0.05) (Table 1).

### 3.2. Comparison of Therapeutic Efficacy between Two Groups

After treatment, 42, 26, and 3 patients in the observation group were assessed as markedly effective, effective, and ineffective, respectively, with an overall effective rate of 95.77%, which was statistically higher than that of 80.60% in the control group (*P* < 0.05). Details are shown in Table 2.

### 3.3. Comparison of Neurological Deficit Scores and Activities of Daily Living between Two Groups before and after Treatment

Before treatment, no statistical differences were observed in NIHSS scores and Barthel index scores between the two groups (*P* > 0.05), while after treatment, NIHSS scores of both groups were decreased and lower in the observation group (11.13 ± 0.97) compared with the control group (17.18 ± 1.16); the Barthel index score increased in both groups and was higher in the observation group (*P* < 0.05). Details are shown in Table 3.

### 3.4. Comparison of the Incidence of Vascular Recanalization, Intracranial Hemorrhage, and Reembolization between Two Groups after Treatment

The vascular recanalization rate after treatment in the observation group was statistically higher than that in the control group, while the incidences of intracranial hemorrhage and reembolization were comparatively lower in the observation group (*P* < 0.05) (Table 4).

### 3.5. Comparison of Serum Inflammatory Factors between Two Groups

**Figure 1:**
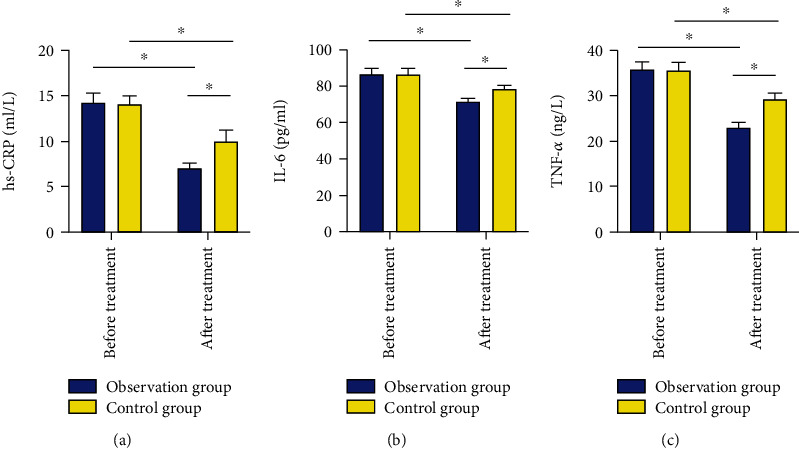
Comparison of serum inflammatory factors between two groups: (a) comparison of serum hs-CRP between two groups of patients; (b) comparison of serum IL-6 between two groups of patients; (c) comparison of serum TNF-*α* between two groups of patients. ^∗^*P* < 0.05.

There were no statistical differences between two groups in terms of IL-6, hs-CRP, and TNF-*α* (*P* > 0.05), while after treatment, these indexes of the observation group were lower compared those of the control group (*P* < 0.05) (Figure 1).

### 3.6. Comparison of Coagulation Function before and after Treatment between Two Groups

**Figure 2:**
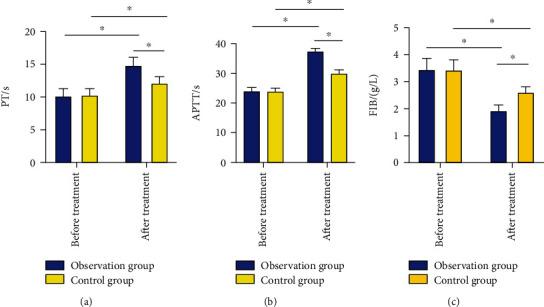
Comparison of blood coagulation function before and after treatment between two groups of patients: (a) comparison of PT between two groups of patients; (b) comparison of APTT between two groups of patients; (c) comparison of FIB between two groups of patients. ^∗^*P* < 0.05.

PT and APTT of two groups were significantly prolonged after 1 week of treatment, while the serum FIB level was statistically decreased (*P* < 0.05); however, compared with the control group, the observation group had longer PT and APTT and a lower level of serum FIB (*P* < 0.05) (Figure 2).

### 3.7. Comparison of the Prognosis between Two Groups of Patients

The modified Rankin Scale was applied to evaluate the prognosis of patients in two groups. The score showed no notable difference between the two groups 1 month after treatment (*P* > 0.05), while decreasing gradually from 3 to 12 months after treatment, with a comparatively lower score in the observation group (*P* < 0.05) (Table 5).

## 4. Discussion

The results of this study showed that intravenous thrombolysis combined with arterial thrombolysis (bridging therapy) could improve the neurological function and daily living ability of patients with ACI and effectively alleviate the inflammatory response of patients. Cerebral infarction is characterized by high disability, recurrence, and mortality rates [14]. The occurrence of cerebral infarction will rapidly induce sensory and motor abnormalities on one side of the patient's limb and even lead to disturbance of consciousness, endangering the life of the patient. Therefore, timely and effective treatment to help patients restore normal vascular circulation is of great significance for reducing the fatality rate and disability rate of this disease and improving patient prognosis [15].

The key to ACI treatment is to open the occluded blood vessels as soon as possible to restore cerebral blood flow in the ischemic penumbra. Ultra-early intravenous thrombolysis and neurovascular intervention are important measures to restore blood perfusion in the infarcted area [16]. Intravenous thrombolysis is currently the main method to open blood vessels in the early stage of cerebral infarction, which can promote local blood circulation through drug dissolution, thus alleviating the ischemia and hypoxia state of brain tissue at the lesion site. Meanwhile, it can speed up the fibrinolysis process and reduce the number of stents used for thrombectomy and the frequency of microvascular thrombus regeneration [17]. However, intravenous thrombolysis alone can easily lead to a low vascular patency rate and a high risk of vascular reocclusion after thrombolysis, which may eventually result in bleeding [9]. Therefore, to restore the recanalization of occluded blood vessels as soon as possible, the medical community has focused on arterial thrombolysis, a treatment that involves direct contact with the thrombus in the blood vessel and complete removal of it by interventional means. Unlike the drug thrombolysis, arterial thrombolysis can remove the thrombus earlier, more successfully, and more completely, thereby greatly improving the early vascular recanalization rate, lowering the risk of recurrence, and significantly improving patients' life quality and prognosis [18].

In the present study, we first compared the daily living ability, neurological function, and therapeutic efficacy between the two groups. The results showed that the observation group had a comparatively more significant improvement in all the above three dimensions on the basis of improvement in both groups. This suggested that patients treated with bridging therapy have better neurological recovery, which is similar to previous studies, indicating that intravenous thrombolysis combined with arterial thrombolysis can remove the thrombus more accurately to play an important role in restoring normal physiological function in the early stage [19]. Then, we compared the incidence of vascular recanalization, intracranial hemorrhage, and reembolization between two groups of patients. It was found that, compared with the control group, the vascular recanalization rate of the observation group was markedly higher, while the incidences of intracranial hemorrhage and reembolization were comparatively lower. Although arterial thrombolysis, as an interventional method, causes certain trauma, the proportion of patients with postoperative complications after bridging therapy did not increase significantly compared with those treated with intravenous thrombolysis alone, suggesting that the combined treatment is safe [20]. Previous studies have found that bridging therapy can safely treat patients with cardiogenic cerebral infarction complicated with anterior circulation macrovascular occlusion, contributing to good vascular recanalization effects and patient prognosis, as well as improved neurological function of patients [21], which corroborates our observations.

Inflammation, which is positively correlated with the severity of cerebral infarction, is essential in the pathogenesis and progression of the disease and acts as an independent pathogenic factor for clinical prognosis and disease outcome assessment of patients [22]. CRP, IL-6, and TNF-*α*, the most representative markers of inflammatory responses, have a direct relationship with cerebral infarction and have gradually become the indicators that researchers and clinical medical staff focus on and attach importance to [23]. Our findings revealed statistically reduced inflammatory factors IL-6, TNF-*α*, and hs-CRP in both groups after treatment, with more obvious decreases in the observation group (*P* < 0.05), which suggested that bridging therapy can relieve inflammatory responses in ACI patients and reduce the high expression of inflammatory factors. It is currently believed that the mechanism leading to thrombosis is mainly the activation of coagulation pathways caused by atherosclerotic plaque rupture, and the whole activating process can be regulated by a variety of coagulation factors [24]. Under the action of various coagulation factors, the body's coagulation function becomes more active, which gradually activates the whole process of thrombosis, resulting in a significant shortening of coagulation time [25]. The results of our study showed that after treatment, the observation group had longer PT and APTT, while having a lower level of serum FIB. This suggested that bridging therapy could better regulate the coagulation function of patients, block the activation of coagulation factors, and prolong the coagulation time than simple intravenous thrombolysis, which better explained our findings. Finally, we analyzed the prognosis of two groups of patients and found that the modified Rankin Scale score of the observation group was markedly lower compared with the control group after 3, 6, and 12 months of treatment. This suggested that bridging therapy can improve the prognosis of patients.

## 5. Conclusion

Intravenous thrombolysis combined with arterial thrombolysis (bridging therapy) can improve the vascular recanalization rate in patients with ACI, reduce the incidence of intracranial hemorrhage and reembolization, and improve patients' prognosis and neurological function, which is worthy of clinical application. However, this study also has certain limitations. First, due to insufficient sample size, the conclusions of the present study need to be further verified. Second, such a study with positive results is still few and flawed, so that prospective randomized controlled studies with a large sample size are still needed to provide more sufficient evidence. Furthermore, efficacy indicators should be evaluated at more time points in future studies to better assess changes in patient prognosis.

## Figures and Tables

**Table 1:** General information [*n* (%)].

Factors	Observation group *n* = 71	Control group *n* = 67	*t*/*χ*^2^	*P*
Gender			0.234	0.629
Male	40 (56.34)	35 (52.24)		
Female	31 (43.66)	32 (47.76)		
Age(years)			0.003	0.956
≤65	30 (42.25)	28 (41.79)		
>65	41 (57.75)	39 (58.21)		
BMI (kg/m^2^)			0.040	0.841
≤23	33 (46.48)	30 (44.78)		
>23	38 (53.52)	37 (55.22)		
History of smoking			0.261	0.609
Yes	37 (52.11)	32 (47.76)		
No	34 (47.89)	35 (52.24)		
Diabetes			0.045	0.833
Yes	32 (45.07)	29 (43.28)		
No	39 (54.93)	38 (56.72)		
Hypertension			0.001	0.980
Yes	38 (53.52)	36 (53.73)		
No	33 (46.48)	31 (46.27)		
Drinking habits			0.084	0.771
Yes	42 (59.15)	38 (56.72)		
No	29 (40.85)	29 (43.28)		

Drinking: drink 5 standard drinking units of alcohol once a week or more.

**Table 2:** Comparison of therapeutic efficacy between the two groups.

Therapeutic efficacy	Observation group *n* = 71	Control group *n* = 67	*t*	*P*
Markedly	42 (59.15)	25 (37.32)	—	—
Effective	26 (36.62)	29 (43.28)	—	—
Ineffective	3 (4.23)	13 (19.40)	—	—
Effective rate	68 (95.77)	54 (80.60)	7.747	0.005

**Table 3:** Comparison of neurological deficit scores and activities of daily living between two groups before and after treatment.

Items	Time	Observation group *n* = 71	Control group *n* = 67	*t*	*P*
NIHSS					
	Before treatment	22.25 ± 1.21	22.21 ± 0.89	0.11	0.913
	After treatment	11.13 ± 0.97	17.18 ± 1.16	33.31	<0.001
Barthel					
	Before treatment	52.15 ± 1.85	52.14 ± 2.2	0.029	0.977
	After treatment	67.36 ± 1.98	59.54 ± 1.62	25.31	<0.001

**Table 4:** Comparison of the incidence of vascular recanalization, intracranial hemorrhage, and reembolization between two groups after treatment.

Items	Observation group *n* = 71	Control group *n* = 67	*χ* ^2^	*P*
Recanalization	64 (90.14)	42 (62.69)	14.59	<0.001
Incidence of intracranial hemorrhage and reembolization	2 (2.82)	10 (14.93)	6.366	0.012

**Table 5:** Comparison of the prognosis between the two groups of patients.

Time	Observation group *n* = 71	Control group *n* = 67	*t*	*P*
1 month after treatment	4.78 ± 0.39	4.83 ± 0.34	0.801	0.425
3 months after treatment	3.62 ± 0.25	4.21 ± 0.29	14.97	<0.001
6 months after treatment	2.39 ± 0.17	3.8 ± 0.26	37.91	<0.001
12 months after treatment	1.8 ± 0.12	2.63 ± 0.17	33.22	<0.001

## Data Availability

The data used to support the findings of this study are included within the article.
